# *Staphylococcus aureus* persistence in osteocytes: weathering the storm of antibiotics and autophagy/xenophagy

**DOI:** 10.3389/fcimb.2024.1403289

**Published:** 2024-06-10

**Authors:** Nicholas J. Gunn, Stephen P. Kidd, Lucian B. Solomon, Dongqing Yang, Eugene Roscioli, Gerald J. Atkins

**Affiliations:** ^1^ Biomedical Orthopaedic Research Group, Centre for Orthopaedic and Trauma Research, Faculty of Health and Medical Sciences, University of Adelaide, Adelaide, SA, Australia; ^2^ Australian Centre for Antimicrobial Resistance Ecology, University of Adelaide, Adelaide, SA, Australia; ^3^ Research Centre for Infectious Disease, School of Biological Science, University of Adelaide, Adelaide, SA, Australia; ^4^ Department of Orthopaedics and Trauma, Royal Adelaide Hospital, Adelaide, SA, Australia; ^5^ Department of Thoracic Medicine, Royal Adelaide Hospital, Adelaide, SA, Australia; ^6^ Department of Medicine, Faculty of Health and Medical Sciences, University of Adelaide, Adelaide, SA, Australia; ^7^ Centre for Cancer Biology, South Australia (SA) Pathology and University of South Australia, Adelaide, SA, Australia

**Keywords:** antibiotic, autophagy, host-pathogen, osteocyte, rifampicin, vancomycin *Staphylococcus aureus*, persister cell

## Abstract

*Staphylococcus aureus* is a major causative pathogen of osteomyelitis. Intracellular infections of resident bone cells including osteocytes can persist despite gold-standard clinical intervention. The mechanisms by which intracellular *S. aureus* evades antibiotic therapy are unknown. In this study, we utilised an *in vitro S. aureus* infection model of human osteocytes to investigate whether antibiotic-mediated dysregulation of autophagy contributes to this phenomenon. Infected or non-infected osteocyte-like cells were exposed to combinations of rifampicin, vancomycin, and modulators of autophagy. Intracellular bacterial growth characteristics were assessed using colony*-*forming unit (CFU) analysis, viable bacterial DNA abundance, and the rate of escape into antibiotic-free medium, together with measures of autophagic flux. Rifampicin, alone or in combination with vancomycin, caused a rapid decrease in the culturability of intracellular bacteria, concomitant with stable or increased absolute bacterial DNA levels. Both antibiotics significantly inhibited autophagic flux. However, modulation of autophagic flux did not affect viable bacterial DNA levels. In summary, autophagy was shown to be a factor in the host–pathogen relationship in this model, as its modulation affected the growth state of intracellular *S. aureus* with respect to both their culturability and propensity to escape the intracellular niche. While rifampicin and vancomycin treatments moderately suppressed autophagic flux acutely, this did not explain the paradoxical response of antibiotic treatment in decreasing *S. aureus* culturability whilst failing to clear bacterial DNA and hence intracellular bacterial load. Thus, off-target effects of rifampicin and vancomycin on autophagic flux in osteocyte-like cells could not explain the persistent *S. aureus* infection in these cells.

## Introduction

*Staphylococcus aureus* is the most common causative pathogen in all forms of adult osteomyelitis, exemplified by that associated with periprosthetic joint infection (PJI). These infections can become refractory to antibiotic therapies through defined resistance mechanisms, or exhibit antibiotic tolerance (in the absence of resistance), by adopting alternative growth phenotypes (Pantosti et al., 2007; Bui et al., 2017). *S. aureus* infection of osteocytes is a feature of osteomyelitis, including in PJI, and *S. aureus* adapts phenotypically to the intracellular environment by forming quasi-dormant small colony variants (SCVs) (Bosse et al., 2005; Tande and Patel, 2014; Yang et al., 2018; Jensen et al., 2023). We recently demonstrated that such variation in the growth state of *S. aureus* following intracellular infection of osteocytes resulted in a discrepancy in bacterial quantification by either colony*-*forming unit (CFU) analysis or quantitative DNA PCR-based approaches, sometimes by several orders of magnitude, likely due to phenotypic switching to a low growth phenotype (Sun et al., 2023). Such phenotypic adaptation is consistent with the high incidence of post-treatment reinfection in PJI, despite antibiotic therapy, being as high as 8.8%–35.5% within 2 years using standard approaches (Kunutsor et al., 2016; Tsang et al., 2017; Malahias et al., 2020; Day et al., 2021; Browning et al., 2022).

Autophagy is a fundamental cellular process with myriad functions related to immunity and cellular homeostasis (Behrends et al., 2010; Tu et al., 2022). Crucially, autophagy is central to the process of degrading and recycling damaged or unnecessary cellular components. LC3 (Microtubule-associated protein 1A/1B-light chain 3) is pivotal in the function of autophagosomes that engulf cellular material targeted for degradation. Initially present in the cytosol as LC3-I, it undergoes lipidation to become LC3-II, which is then recruited to the growing autophagosomal membrane. This recruitment is essential for autophagosome elongation and closure and facilitates cargo sequestration within the autophagosome. Sequestosome 1 (SQSTM1), also known as p62, acts as a selective autophagy receptor, recognising ubiquitinated cargo destined for degradation and linking it to LC3-II on the autophagosomal membrane. Xenophagy is a specialised form of autophagy that specifically eliminates invading pathogens, such as bacteria, viruses, and parasites. Degradation of the autophagosomal cargo is mediated by fusion with lysosomes to form the autophagolysosome (King, 2012; Mao and Klionsky, 2017; Mitchell and Isberg, 2017; Riebisch et al., 2021).

There are few situations in which autophagy/xenophagy is subject to clinical or diagnostic inquiry, despite the critical role played by this pathway in host cell clearance of intracellular infections. Given that pathogenic bacteria have evolved means to subvert, disrupt, or even make use of components of the autophagic/xenophagic pathway to reside intracellularly, it is important that antibiotics do not contribute to this outcome (Rubinsztein et al., 2012). We have shown that antibiotics can potently arrest innate intracellular bacterial clearance processes in lung epithelium (Poh et al., 2020). Several antibiotics used for the treatment of Staphylococcal PJI (Zelmer et al., 2022) have been identified to elicit this off-target effect (Tande and Patel, 2014). For example, the aminoglycoside gentamicin, which is commonly used in antimicrobial-loaded bone cements, has been demonstrated to inhibit autophagosome–lysosome fusion, an effect that is also mediated by intracellular *S. aureus* (Schnaith et al., 2007; Kim et al., 2017). Other aminoglycosides, such as tobramycin, aggregate and inhibit lysosomal phospholipase activity when fully protonated within the acidic environment of lysosomes (Mingeot-Leclercq and Tulkens, 1999). In addition, vancomycin-induced kidney injury appears to be at least partly mediated by autophagic dysregulation, via its accumulation and subsequent rupture of autophagolysosomes (Pais et al., 2020). Vancomycin has also been demonstrated to block autophagic flux in macrophage cell lines (Ha et al., 2015). Rifampicin, on the other hand, has been shown to inhibit rapamycin-induced autophagy through the suppression of the positive regulator of autophagy, PP2A (Park et al., 2008).

Taken together, these observations point to a scenario whereby the efficacy of antibiotics in clearing extracellular infection may mask undesirable off-target effects on autophagy/xenophagy that promote intracellular residence of *S. aureus*. In the current study, we sought to determine the involvement of autophagy in a human osteocyte model of *S. aureus* infection and the impact of two commonly co-administered antibiotics, rifampicin and vancomycin, on this interaction. We found significant interruption of normal autophagy by both antibiotics, but this may not be consequential to the capacity of *S. aureus* to persist within osteocytes.

## Materials and methods

### Cell culture

Cell culture of SaOS2 cells and differentiation under osteogenic growth conditions to an osteocyte-like stage (SaOS2-OY) were performed as previously reported (Prideaux et al., 2014; Gunn et al., 2021). Briefly, SaOS2 cells were seeded at 1.5 × 10^4^ cells per well of a 48-well tissue culture plate and cultured for 28 days in differentiation medium consisting of αMEM (Gibco, NY, USA) with 5% v/v foetal calf serum, 10 mM HEPES, 2 mM L-glutamine (Thermo-Fisher, VIC, Australia), and 50 µg/mL ascorbate-2-phosphate and 1.8 mM potassium di-hydrogen phosphate (Sigma, MO, USA). Media were refreshed twice weekly during the 28-day differentiation period and weekly thereafter. During differentiation, the medium was supplemented with penicillin/streptomycin (Thermo-Fisher, VIC, Australia), both at 1 unit/mL. This protocol results in confluent differentiated cultures containing approximately 1.2 × 10^5^ cells/well, as recently determined using a genome quantification protocol (Sun et al., 2023). Following infection, where relevant, media were either antibiotic-free or supplemented with 10 µg/mL lysostaphin (AMBI Products LLC, NY, USA). Routine light microscopy showed that the differentiated osteocyte-like cultures exhibited the characteristic mineralised extracellular matrix consistent with this model (Prideaux et al., 2014; Gunn et al., 2021).

### Bacterial culture and SaOS2-OY infection

Growth and quantification of the methicillin-resistant *S. aureus* (MRSA) strain, WCH-SK2, was performed as previously described (Gunn et al., 2021). This strain was chosen due to its demonstrated ability to establish intracellular infections in human osteocyte-like cells and in so doing rapidly alter their growth phenotype, in particular switching to an SCV state (Yang et al., 2018; Gunn et al., 2021). Briefly, bacteria were plated onto nutrient broth agar (NBA) from glycerol stocks to ensure the culture was a monoculture of the intended strain. Isolated colonies were sub-cloned twice into nutrient broth. Log-phase broth was pelleted (2,000 × *g* for 10 min at ambient temperature) and resuspended in phosphate buffered saline (PBS) to approximate the desired target CFU/mL from a standard curve of absorbance at OD_600nm_.

Infection of SaOS2-OY cultures was performed, as previously described (Gunn et al., 2021). Briefly, differentiation medium was removed from SaOS2-OY cultures and the wells were washed twice with PBS before the addition of the suspended bacteria. The actual CFU/mL was confirmed by plating serial dilutions of the inoculate onto NBA. Following a 2-h infection period, the wells were washed twice with PBS and exposed to medium containing 10 µg/mL lysostaphin for 2 h to clear extracellular bacteria. For “acute infection” experiments, media were replaced with fresh media containing the described treatments. For “chronic infection” experiments, cultures were maintained in media containing lysostaphin for a period of 8 days, unless otherwise stated, whereupon cell lysates were confirmed to be non-culturable prior to commencement of treatments. Antibiotic treatments were performed, as described below. In addition, for some experiments, modulators of autophagy Ku-0063794 [KU; autophagy inducer (Nyfeler et al., 2012); Sigma-Aldrich] at 10 μM and bafilomycin (autophagy blocker; Jomar, VIC, Australia) at 10 nM were tested for their respective effects on bacterial and autophagic outcomes. For acute infection experiments, SaOS2-OY cultures were pre-treated with each modulator for 4 h prior to the addition of bacteria. For chronic infection experiments, these agents were added 24 h prior to each readout.

### Quantification of bacterial burden by qPCR

For intracellular bacterial retrieval, host cells were lysed in sterile water for 20 min at 37°C. Where exclusively viable bacterial DNA was required, the lysate was treated with the photoreactive DNA-binding dye PMAxx (Biotium, CA, USA). Briefly, PMAxx was added to lysates at a final concentration of 20 µM, and this was incubated for 10 min at 37°C under blue light. After this, DNA was extracted using the Gram-positive bacterial cell lysate protocol as per the PureLink™ genomic DNA mini kit (Thermo-Fisher), except that the suggested lysozyme was replaced with 20 µg/mL lysostaphin. Real-time qPCR was performed to quantify relative bacterial gene abundance using RT^2^ SYBR Green Fluor qPCR Mastermix (Qiagen, Limburg, Netherlands) and the CFX Connect thermocycler (Bio-Rad, CA, USA). Where appropriate, bacterial DNA quantity was normalised to the magnitude of human *ACTB* DNA using the 2^−ΔCT^ method. When PMAxx treatment was used, as it inhibits extraction of human DNA from the lysates, human DNA abundance was determined from replicate cultures extracted in the absence of PMAxx. The sequences of the oligonucleotide primer sets used are listed in **Supplementary Table 1**.

### Measurement of SaOS2-OY cell viability

Cell viability was assessed using the lactate dehydrogenase (LDH) enzyme activity method (Cytotoxicity Detection Kit version 11; Sigma-Aldrich) to quantify compromised cells in response to exposure to antibiotics. SaOS2-OY cultures seeded into 48-well tissue culture plates were treated with a dose range of either rifampicin (0.75–75 µg/mL) or vancomycin (3–800 µg/mL) diluted in differentiation medium. LDH activity quantification was performed 24 h after the initiation of treatments, following the manufacturer’s protocol.

### Measurement of culturable bacteria per well

Bacterial outgrowth was routinely monitored by observation of media colour change and turbidity. For experiments where bacterial outgrowth was an outcome, this was further measured by daily plating of 1.5 µL of media onto NBA. For the purpose of measuring culturable intracellular bacteria per well, after thorough rinsing in sterile PBS to remove antimicrobial agents, SaOS2-OY cells were lysed by 20-min exposure to pure water at 37°C. Following lysis, the cell layer was scraped into solution and agitated by repeated passage through a 200-µL pipette tip. The resulting clumped cell debris was separated by centrifugation from the lysate, which was serially diluted 1/10 in sterile PBS and 100 μL plated onto NBA and incubated at 37°C. CFUs were counted after 48 h of incubation. Growth was monitored for a minimum of a further 14 days to ensure that all culturable bacteria had time to generate macroscopically visible colonies.

### Antibiotic treatments

When analysing and comparing the effects of antibiotics on bacterial outcomes, we used minimum bactericidal concentrations (MBC) for planktonic growth to normalise the comparison. These were determined by octuple replicate culture of log-phase bacteria into fresh nutrient broth containing a dose range of the antibiotic in question. The lowest dose for which no growth occurred following 24 h of culture was designated as the MBC. Culture was confirmed by drop plating from the broth onto NBA. For vancomycin, the planktonic MBC for *S. aureus* WCH-SK2 was measured to be 8 µg/mL. The equivalent concentration for rifampicin was 0.75 µg/mL. When interrogating the effects of antibiotics on the host cells, we used concentrations that included the 1× MBC to maintain intra-study comparisons and doses that approximate clinically relevant ranges. We ensured that the ranges were proportionate to each other to allow comparisons to be made. For vancomycin, in this context, we used 3, 8, and 20 µg/mL whilst osteocyte exposure ranges are predicted to be 1.1–6 µg/mL and serum trough concentrations for *S. aureus* infections are recommended to be 15–20 µg/mL (Rybak et al., 2009; Zelmer et al., 2022). The equivalent range used herein for rifampicin was 0.75, 2.5, and 6 µg/mL. The concentrations osteocytes are expected to be exposed to are 1.3–6.5 µg/mL (Zelmer et al., 2022). Antibiotics were added only at the beginning of each time course indicated.

### Quantification of autophagic flux

Protein was isolated from differentiated cultures using M-PER™ mammalian cell protein lysis reagent and Halt^®^ protease and phosphatase inhibitor cocktail (both from Thermo Scientific, Victoria, Australia). Protein samples were quantified using the BCA protein assay method (Bio-Rad, Victoria, Australia), and 10 μg (unless specified otherwise) were electrophoresed using Novex^®^ 4%–12% gradient Bis-Tris denaturing gels (Life Technologies, Victoria, Australia) and electroblotted to Trans-Blot^®^ Turbo nitrocellulose membranes (Bio-Rad). Membranes were blocked in 5% diploma skim milk for 1 h and then probed overnight at 4°C with primary antibodies directed to LC3A/B I-II (# 4108 in 5% BSA), Sequestosome (#5114 in 5% skim milk; both Cell Signaling Technology, Boston, MA), and β-actin (#A1978 in 5% skim milk; Sigma-Aldrich Co.) followed by matching horseradish peroxidase-conjugated secondary antibodies for 1 h at room temperature (R&D Systems, MN, USA). Chemiluminescent imaging was performed using the LAS-4000 platform and histogram densitometry was performed using Multi Gauge software (V3.1 Fujifilm, Tokyo, Japan). Density scores were normalised to the β-actin control and analysis was performed consistent with relevant guidelines (Loos et al., 2014; Yoshii and Mizushima, 2017).

### Transmission electron microscopy

Uninfected SaOS2-OY cells were prepared for transmission electron microscopy (TEM), as previously described (Yang et al., 2018; Gunn et al., 2021). Briefly, they were cultured in 75-cm^2^ cell culture flasks before a 24-h, 37°C exposure to fixative containing 1.25% v/v glutaraldehyde, 4% w/v sucrose, and 4% w/v paraformaldehyde in PBS. After fixation, the cells were demineralised to facilitate sectioning in Osteosoft™ solution (Sigma-Aldrich). The fixed and demineralised cells were processed for imaging in 2% w/v osmium tetroxide before being embedded in resin and cut into 5-µm sections.

### Intracellular infection escape assay

In order to assess resuscitation of intracellular *S. aureus* infections, a bacterial outgrowth or escape assay was generated. SaOS2-OY in 48-well plates were infected with a dilute suspension of WCH-SK2 (0.05 MOI; in PBS). The infection was maintained for 2 weeks in a solely intracellular state under the pressure of lysostaphin-supplemented differentiation medium, as reported (Gunn et al., 2021), to shift the bacterial community into a chronic-like, predominantly non-culturable state. At 15 days post-infection, media were replaced with antimicrobial-free differentiation media containing the modulators of autophagy Ku-0063794 (10 µM) or bafilomycin (10 nM), or untreated control media. Additionally at this time, host cell lysates were assessed for (non-)culturability. Following the initiation of treatment, bacterial content was assessed daily (excluding 6 days post-treatment) by drying 2 µL/well of supernatant onto NBA to ascertain whether bacteria with a culturable phenotype were present in the media.

### Statistical analyses

All statistical analyses were performed using GraphPad Prism software v. 9.0.0 (GraphPad Prism, La Jolla, CA, USA). Differences in CFU counts over time were tested using one-way ANOVA with Tukey’s *post-hoc* test. Antibiotic dose responses analysed by Western blot were analysed by simple linear regression. Protein levels in response to various treatments were tested by the Brown–Forsythe ANOVA test with unpaired *t* with Welch’s correction multiple comparisons test. Antibiotic dose effects on cell viability analysed by LDH assay were analysed by simple linear or non-linear regression and plotted with 95% confidence intervals. Outgrowth well survival curves were compared with the Log-rank Mantel–Cox test. In all cases, a value for *p* < 0.05 was considered significant.

## Results

### Effects of rifampicin and vancomycin on *S. aureus* intracellular culturability and bacterial burden

**Figure 1:**
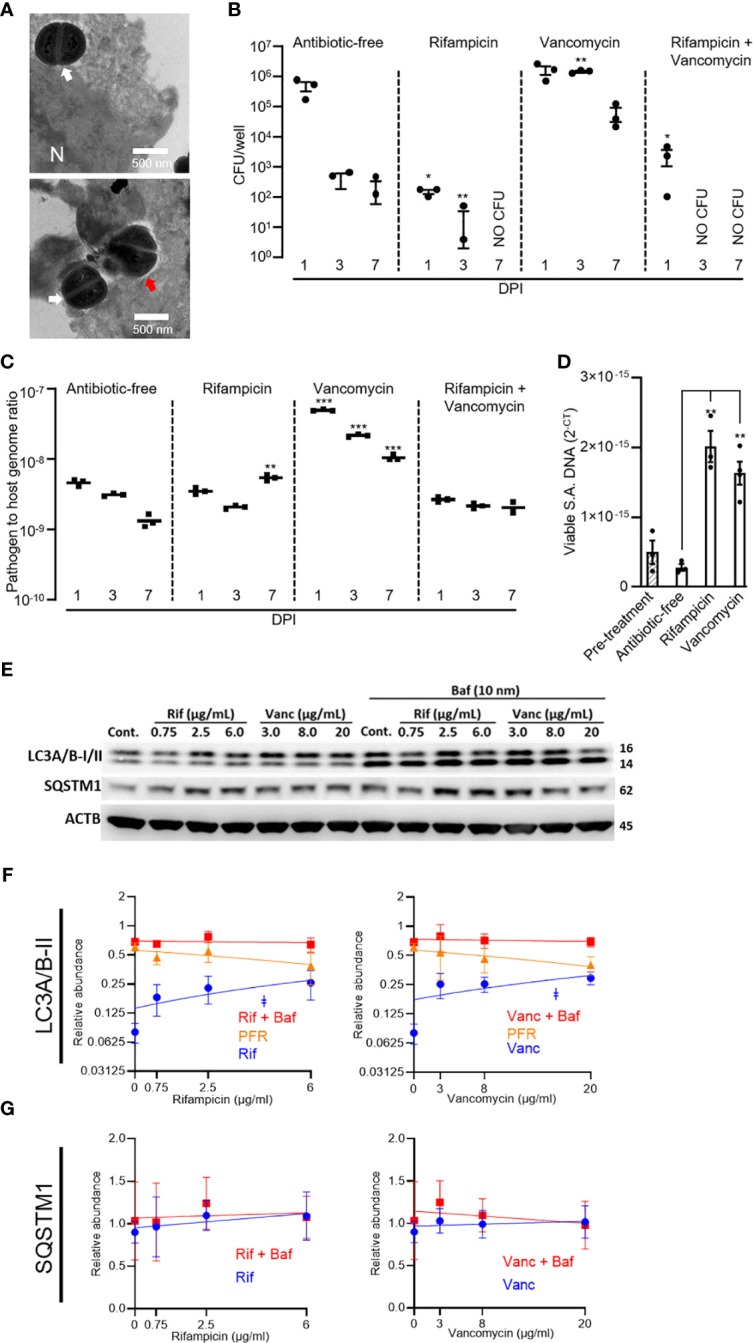
Effects of rifampicin and vancomycin on infection dynamics and autophagy in the SaOS2-OY model. SaOS2-OY cultures were infected with WCH-SK2 at an MOI of 1. **(A)** TEM analysis at 3 h post-infection (N = nucleus; white arrow: dividing S. aureus; red arrow: degrading S. aureus). Cultures were either untreated or treated with 1 × MBC of rifampicin (0.75 µg/mL), 1 × MBC of vancomycin (8 µg/mL), or a combination of both antibiotics at their respective MBC. **(B)** CFU counts [mean ± standard deviation (SD)] from lysates of triplicate wells at 1, 3, and 7 days DPI ± antibiotic treatments were measured. Differences between treatments and antibiotic-free controls were determined using Student’s *t*-tests; **(C)** Half of the lysate from each well was pooled within groups for total DNA isolation following treatment with PMAxx™. Bacterial DNA abundance was measured with qPCR for sigB against host ACTB DNA levels and expressed as 2*^−^* ^ΔCT^ (mean ± SD). **(D)** Effects of antibiotics in a chronic infection model were also tested in infected cultures, as above, but maintained in lysostaphin-supplemented media until host cell lysates yielded no CFU (17 DPI). Cultures were then exposed to antibiotic-supplemented (1× MBC, as above) or control media for 24 h. Host cell lysates in triplicate were treated with PMAxx and assayed for viable sigB DNA by qPCR and expressed as 2*^−^* ^CT^ (mean ± SD). Relative LC3A/B-II quantity per well was tracked across clinically relevant respective dose ranges of rifampicin and vancomycin. Measurements were taken at 6 h following treatment initiation ± bafilomycin. **(E)** Representative Western blot. Relative abundance of **(F)** LC3A/B-II and **(G)** p62/SQSTM1 protein. Significance (indicated by ǂ p < 0.05) associated with the linear regressions indicates whether the slopes of the relationships were non-zero. Potential flux remaining (PFR) was determined by subtracting treatment values from the treatment + bafilomycin values. Data are mean ± SD of biological quadruplicates. DPI, days post-infection; CFU, colony-forming units; MBC, minimum bactericidal concentration; SA, *S. aureus*; Cont., control; Rif, rifampicin; Vanc, vancomycin; Baf, bafilomycin. Significant difference to controls is indicated by *p < 0.05, **p < 0.01, ***p < 0.001.

*S. aureus* intracellular infections, using the MRSA strain WCH-SK2, were established in SaOS2-OY cells. Consistent with our published observations with this model (Gunn et al., 2021), examination by TEM revealed both viable and dividing intracellular *S. aureus*, as well as bacteria in the process of being cleared by autophagolysosomal degradation (**Figure 1A**). Cultures were exposed to rifampicin and/or vancomycin at 1× the respective MBC. In the absence of antibiotics, the SaOS2-OY model demonstrated the capacity to clear a significant proportion of intracellular bacteria over the course of the observation interval with a time-dependent decrease in recovered intracellular CFU (**Figure 1B**). Rifampicin treatment was effective in clearing culturable intracellular bacteria, and this effect was heightened with co-exposure to vancomycin. Vancomycin treatment alone was ineffective at reducing bacterial burden and rather significantly increased CFU number at 3 DPI. We next measured intracellular bacterial DNA levels as a surrogate for total bacterial numbers; in order to exclude molecular detection of dead bacteria, the photoreactive viability PCR reagent, PMAxx™, was used. Despite effects on CFU, neither antibiotic alone or in combination reduced relative intracellular bacterial DNA levels compared to untreated controls (**Figure 1C**), suggesting bacterial persistence in the face of these treatments. Rifampicin caused a significant retention or increase in *S. aureus* DNA at 7 days compared to antibiotic-free controls, whilst vancomycin alone caused a significant increase in bacterial DNA levels at all time points (**Figure 1C**). Interestingly, bacteria recovered from rifampicin-treated cells displayed rifampicin resistance whilst those that grew from vancomycin-treated cells retained vancomycin sensitivity (data not shown).

The potential of the antibiotics to influence the frequency of persistent intracellular *S. aureus* was further assessed in a long-term “chronic” infection model. For this, SaOS2-OY cells were infected with *S. aureus* WCH-SK2 and then cultured in the continued presence of lysostaphin until measures of intracellular (and extracellular) CFU were below detection, which occurred at 17 days post-infection. Following this, antibiotic treatments were applied for 24 h. **Figure 1D** shows that non-culturable viable bacterial abundance was appreciably increased within the SaOS2-OY cells challenged with either antibiotic compared with controls, consistent with findings in the acute model.

### Effects of rifampicin and vancomycin on autophagic flux in osteocyte-like cells

To examine possible off-target effects of antibiotics on bacterial clearance mechanisms, SaOS2-OY cells were exposed to clinically relevant dose ranges of rifampicin or vancomycin and assessed for alterations of autophagic flux. The SaOS2-OY model demonstrated significant autophagic potential, as indicated by the differential LC3A/B-II signal [corresponding to the lower 14 kDa band (Mizushima and Yoshimori, 2007)] for the control exposure (i.e. basal LC3A/B-II) and its accumulation with the autophagy inhibitor, bafilomycin (+2.76-fold/flux of LC3A/B-II for bafilomycin vs. no-exposure, β-actin normalised, *p* = 0.025, *n* = 3, **Figures 1E, F**). The abundance of LC3A/B-II positively correlated with increasing concentrations of rifampicin and vancomycin (**Figure 1E**, *p* = 0.044 and *p* = 0.047, respectively) and appeared to plateau at a similar proportion of maximal flux inhibition (indicated by levels in the co-presence of bafilomycin), 34% and 37%, respectively. While apparent accumulation of p62/SQSTM1 was also observed (**Figure 1E**), the repeat outcomes did not achieve statistical significance, likely due to an already relatively high basal abundance, which was not altered even in the added presence of bafilomycin (**Figure 1G**). While rifampicin displayed cytotoxicity at doses above 25 µg/mL, neither antibiotic impaired cell viability in the dose ranges utilised for protein-based or cell culture experiments (**Supplementary Figure 1**).

### Effect of autophagic flux modulation in an acute infection model

**Figure 2:**
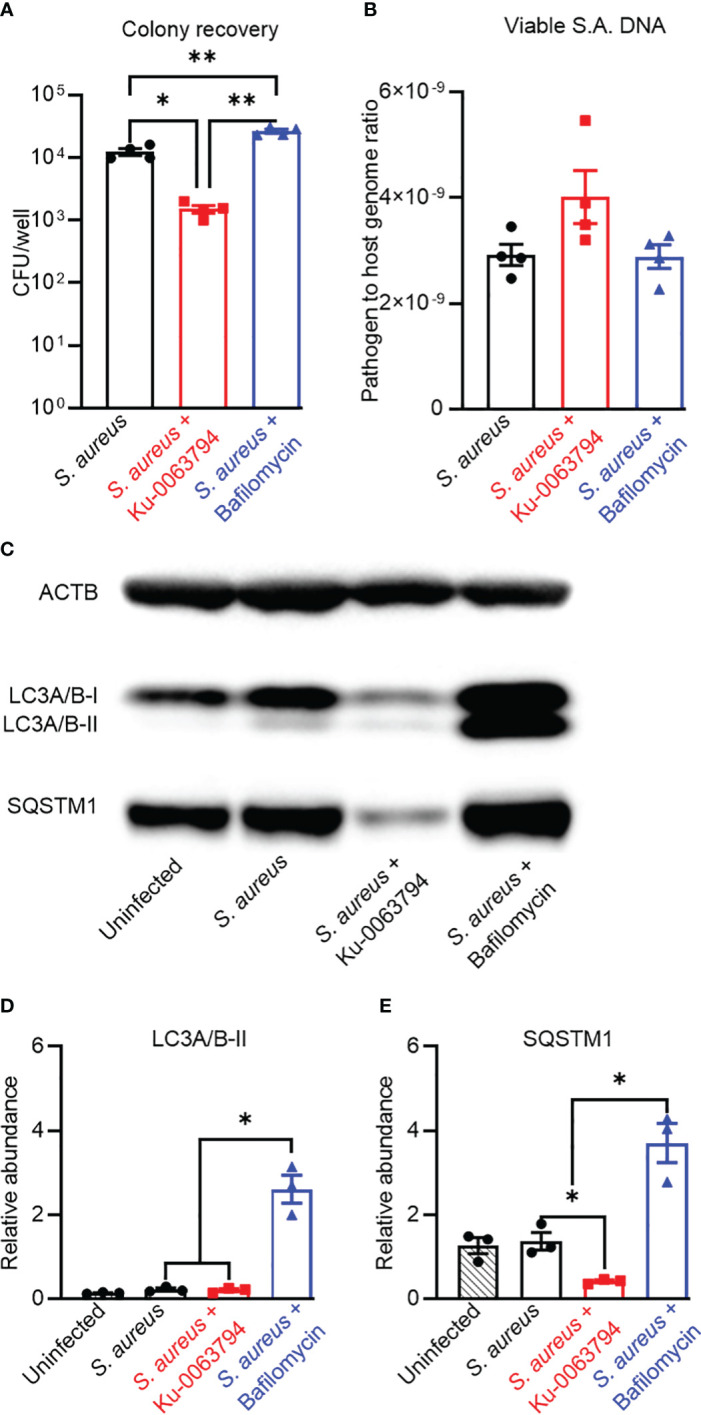
Analysis of the impact of autophagic flux promoters or blockers on bacteriological and autophagic outcomes in an acute infection context. Differentiated SaOS2-OY cells were pre-treated with either control media (no treatment) or with media containing either Ku-0063794 (10 µM) or bafilomycin (10 nM) for 4 h. then exposed to WCH-SK2 at MOI = 1, as described in Materials and Methods. After the 2-h infection period, treatments were replenished in media also containing lysostaphin to clear extracellular bacteria and cells cultured for a further 24 h before assaying for **(A)** colony-forming units (CFU) per well from host cell lysates. Data are mean ± SD of biological quadruplicates; **(B)** relative sigB DNA expressed as 2*^−^* ^ΔCT^ from host cell lysates treated with PMAxx™ reagent prior to DNA purification; data are mean ± SD of biological triplicates. **(C)** Representative image of a Western blot (*n* = 3) used for protein quantification. **(D)** Relative LC3A/B-II protein quantity per well from host cell lysates. **(E)** Relative p62/SQSTM1 quantity per well from host cell lysates. Protein levels were normalised to ACTB. Data are mean ± SD of biological triplicates. Significant difference to controls is indicated by *p < 0.05 and **p < 0.01.

Given the confirmation of antibiotic inhibition of autophagy, we next examined whether the autophagy apparatus and *S aureus* interact within the context of an acute infection model. When SaOS2-OY cultures acutely infected with *S. aureus* WCH-SK2 were subjected to exogenous potentiation of autophagic flux with Ku-0063794 (inhibitor of mTORC1 and mTORC2), there was a significant 87.9 ± 3.3% reduction relative to the control in the recoverable intracellular CFU/well (**Figure 2A**, *p* = 0.015). In line with this observation, blockade of autophagy with bafilomycin resulted in a 2.1 ± 0.3-fold increase in the number of CFU within the host cell lysates (**Figure 2A**, *p* = 0.004). However, neither autophagy modulator altered the bacterial genomic *sigB* signal (**Figure 2B**).

Western blot analysis of protein lysates from these cultures confirmed modulation of autophagy in an infection context (**Figures 2C–E**). Infected controls exhibited slight, albeit non-significant, blockage of autophagy based on visually elevated LC3-II and p62/SQSTSM1. Strong induction of autophagic flux in infected cells was seen with Ku-0063794, which resulted in significantly reduced p62/SQSTSM1 (*p* < 0.05). Flux potentiation also resulted in apparent reduced LC3A/B-II abundance relative to the infected control, but this did not achieve significance, likely due to the already low starting level. Bafilomycin treatment resulted in the concomitant accumulation of these markers of autophagic flux, consistent with its inhibition.

### Effect of autophagic flux modulation in a chronic infection model

**Figure 3:**
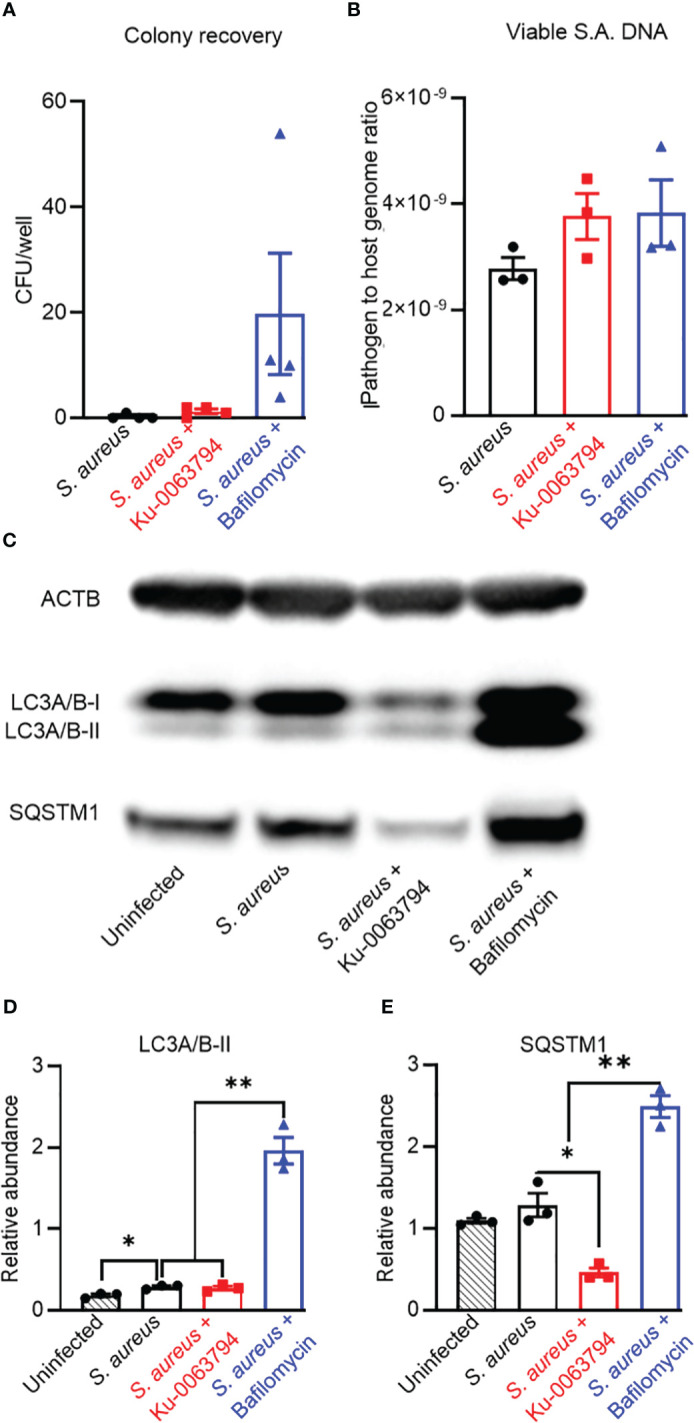
Analysis of the impact of autophagy modulators on bacteriological and autophagic outcomes in a chronic intracellular infection context. Differentiated SaOS2-OY cells were infected at an MOI of 1 with WCH-SK2, as described in Materials and Methods. Cells were cultured for 7 days and cell lysates were confirmed to yield no CFU on NBA after 24 h. Cultures (at day 8) were then treated with either control media (no treatment) or with media containing either Ku-0063794 (10 µM) or bafilomycin (10 nM) for a further 24 h before assaying for **(A)** CFU per well from host cell lysates. Data are mean ± SD of biological quadruplicates; **(B)** relative sigB DNA expressed as 2^−ΔCT^ from host cell lysates treated with PMAxx™ reagent prior to DNA purification; data are mean ± SD of biological triplicates. **(C)** Representative image of a Western blot (*n* = 3) used for protein quantification. **(D)** Relative LC3A/B-II protein quantity per well from host cell lysates. **(E)** Relative p62/SQSTM1 quantity per well from host cell lysates. Protein levels were normalised to ACTB. Data are mean ± SD of biological triplicates. Significant difference to controls is indicated by *p < 0.05 and **p < 0.01.

We next investigated the influence of autophagy modulation in a chronic infection model, where the infected cultures first exhibited intracellular bacteria with a uniformly non-culturable phenotype prior to treatment, i.e. at 8 days post-infection. Promotion of autophagy in chronically infected cultures with Ku-0063794 maintained the culturable and viable population to a similarly low level as the control exposure (*p* = 0.26). However, inhibition of autophagy with bafilomycin resulted in a small but significant increase in culturable intracellular bacteria, consistent with autophagy actively clearing *S aureus* during the observation interval (**Figure 3A**). Similar to the acute model, neither treatment affected *S. aureus* intracellular DNA levels (**Figure 3B**). Indeed, based on relative DNA levels, approximately the same frequency of an apparent persistent subpopulation of *S. aureus* remained between these models, despite the increased duration of infection (**Figure 2B** vs. **Figure 3B**). Infected controls displayed blockage of autophagic flux with significantly elevated LC3A/B-II (**Figures 3C, D**). Potentiation of autophagy resulted in a clear reduction in p62/SQSTM1, whilst blockage with bafilomycin increased both LC3A/B-II and p62/SQSTM1 levels (**Figures 3C–E**). Together, these data suggest a continued and increased disassociation between autophagic flux modulation and *S. aureus* persistence in a chronic infection.

### Effect of autophagic flux modulation on bacterial release

**Figure 4:**
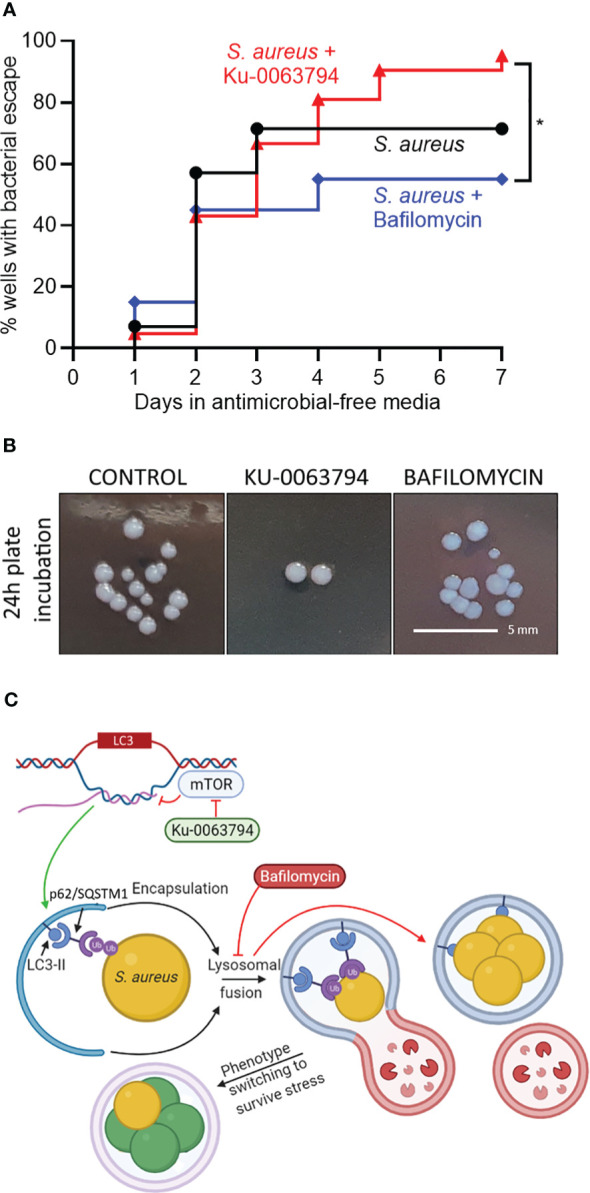
Effect of autophagic modulation on bacterial escape from the intracellular niche. Differentiated SaOS2-OY cells were infected at an MOI of 1 with WCH-SK2, as described in Materials and Methods. Cells were cultured in medium containing lysostaphin for 15 days and cell lysates were confirmed to yield no CFU after 24 h. Medium was then replaced with either control medium (no treatment) or with that containing either Ku-0063794 (10 µM) or bafilomycin (10 nM), all in the absence of any other antimicrobial treatment, and cultures (at least 20 wells/treatment) were monitored by visual inspection for bacterial outgrowth for a further 7 days. **(A)** Inverted Kaplan–Meier estimator tracking the percent of wells with outgrowth. **(B)** Gross colony morphological assessment from each experimental group after 1 day of plate incubation. Statistical differences between survival curves were analysed with a log-rank test and results are indicated by *p < 0.05. **(C)** Schematic of the proposed interaction of Ku-0063794 and bafilomycin-mediated modulation of autophagy on intracellular *S. aureus* within SaOS2-OY cells. LC3 is given as an example of an autophagic gene whose transcription is modulated by mTOR blockade. Following autophagosomal entrapment and LC3A/B-II-mediated formation of the autophagolysosome, *S. aureus* phenotype switching occurs (*green* cells), resulting in a decline in culturability on nutrient agar, although with a subpopulation of persister cells (*gold*) with potential to revert to a wild-type growth state, such that there is no significant change in bacterial population size. This process is promoted through xenophagic potentiation with Ku-0063794. Treatment with bafilomycin prevents lysosomal fusion promoting a culturable phenotype. Ub, Ubiquitin. Image created using BioRender.com.

To further test the involvement of autophagic flux in bacterial persistence in osteocytes, we designed a bacterial outgrowth assay, where a long-term (17 day) non-culturable infection was treated with autophagy modulators in antimicrobial-free media. The outcome measured was detectable bacterial growth in culture supernatants, analogous to relapse of a chronic infection. This was scored as the percentage of wells containing bacterial outgrowth over time. Autophagic potentiation with Ku-0063794, when compared to blockade with bafilomycin, increased the rate of bacterial escape and growth over time, with the divergent effect becoming evident after approximately 4 days post-treatment (**Figure 4A**, *p* = 0.03). Media from all wells with outgrowth generated near-confluent growth on agar plates. Where single colonies were discernible, no differences in colony morphology was seen (**Figure 4B**): all groups presented with unpigmented colonies after 24 h of plate incubation, which reverted to gold pigmentation and haemolytic capability upon further incubation, typical of *S. aureus* wild-type growth characteristics.

## Discussion

Consistent with our previous reports, human osteocyte-like SaOS2-OY cells displayed the capacity to clear a significant proportion of internalised *S. aureus* in the absence of exogenous intervention (Yang et al., 2018; Gunn et al., 2021), suggesting an active autophagy/xenophagy system in these cells. However, *S. aureus* clearance is incomplete, with evidence of persistence of bacterial DNA within the host cell, despite a decline in culturable bacterial numbers (Sun et al., 2023). As the infection of osteocytes by *S. aureus* is proposed to represent an important niche of persistence within bone in PJI (Yang et al., 2018; Urish and Cassat, 2020), we sought to test to what extent, if any, this process was dependent on manipulation of the autophagy/xenophagy pathway. At the same time, it was important to establish if clinically utilised antibiotics were effective in this model and if off-target effects on autophagic flux influenced the treatment. Whilst rifampicin alone and in combination with vancomycin was effective in reducing recoverable intracellular CFU, we found that neither antibiotic cleared intracellular bacteria as measured by viable DNA levels, and in fact increased this measure in a chronic infection model where there was a complete absence of culturable bacteria. Neither vancomycin or rifampicin were able to eliminate viable intracellular *S. aureus* at the respective MBCs for the strain tested under planktonic conditions, which fall within the ranges we expect osteocytes to be exposed to *in vivo* during clinical use (Zelmer et al., 2022). This was not altogether surprising, given the additional protection the intracellular environment provides the invading pathogen. Vancomycin targets cell wall synthesis and is generally effective against *S. aureus* infections. However, its intracellular effectivity has generally been found to be poor, due in part to its slow accumulation and possibly also the natural resistance of slow growth phenotypes to this killing modality (Zelmer et al., 2022). Rifampicin, on the other hand, has excellent intracellular penetrance and inhibits bacterial transcription, although its effectivity against intracellular *S. aureus* appears to be strain-dependent (Zelmer et al., 2022). It is interesting that, whilst there were differences in the efficacy of the two antibiotics at reducing culturability, both were capable of causing an increase in the total bacterial population size in a manner that would be overlooked by current routine clinical diagnostic procedures, even though they operate through entirely distinct mechanisms.

We showed recently that enumeration of *S. aureus* by culture alone in an intracellular context lacks accuracy, certainly in human osteocytes (Sun et al., 2023) but likely also in other host cell types. *S. aureus* has been shown to replicate intracellularly in the endosomes of various cell types, including THP-1 macrophages and HUVECs (Fraunholz and Sinha, 2012). Consequently, our observations suggest that antibiotic-driven bacterial adaptation facilitates greater rates of replication within the intracellular niche or alternatively that an effect of the antibiotics is to inhibit host cell clearance of bacteria or inhibit their replication.

The presence of a non-culturable but metabolically active persister phenotype of *S. aureus* was evidenced by the relative increase in bacterial DNA within intact bacteria following treatment with both rifampicin and vancomycin, as well as the ability of a chronically non-culturable infection to spontaneously revert to a culturable state when antimicrobial pressure was removed. Whilst the exact nature of these persister-like cells was not characterised herein, this reflects the viable but non-culturable state often described in the context of biofilm formation (Tuchscherr et al., 2016; Ayrapetyan et al., 2018).

The effects of either antibiotic could not be explained by cytotoxicity at the doses tested. However, both antibiotics caused an acute, significant dose-dependent inhibition of autophagic flux at their respective clinically relevant doses. Given the observed antibiotic-mediated increase in LC3A/B-II, the underlying mechanism is likely at least partial inhibition or disruption of lysosomal maturation or autophagolysosomal degradation or fusion, similar to the effects of the definitive autophagy blocker, bafilomycin (Mauvezin and Neufeld, 2015). While the apparent lack of modification of p62/SQSTM1 quantity by bafilomycin treatment in uninfected SaOS2-OY cells implies that this adaptor protein is not involved in the basal flux being investigated, *SQSTM1* is known to be highly transcriptionally regulated (Zhong et al., 2016) and a subsequent effect on protein levels may have been missed due to the time intervals examined.

Lysosomal maturation and autophagosome–lysosome fusion represent key steps in the xenophagic pathway, which facilitates the clearance of intracellular bacteria, and *S. aureus* has previously been shown to be capable of inducing the formation of a replicative niche through the dispruption of these processes in other cell contexts (Neumann et al., 2016; Geng et al., 2020). *S. aureus* indeed induced some degree of autophagic flux inhibition in our osteocyte infection model, best evidenced by the increased level of LC3A/B-II in the chronic model (see **Figure 3**). Interestingly, p62/SQSTM1 was not significantly increased by infection itself. Potential explanations for this observation are functional redundancy via other autophagy adaptors, a proportional increase in the rate of p62/SQSTM1 degradation, additional effects on transcriptional regulation of *SQSTM1* (Zhong et al., 2016) masking changes in protein levels, or a yet-to-be-identified effect exerted by the infecting bacteria. Nonetheless, p62/SQSTM1 appeared to retain its function, as the autophagy potentiators and blockers were capable of significantly altering relative p62/SQSTM1 quantities when bacteria were present (see **Figures 2**, **3**). We sought to investigate whether the paradoxical decrease in culturability but retention or increase in bacterial DNA levels observed with antibiotic treatment was explainable by autophagy dysregulation. As such, we tested the effects of bafilomycin and the autophagic flux potentiator Ku-0063794 on microbiological outcomes in intracellularly infected cells. Neither treatment when applied either immediately following infection or after chronicity was established resulted in a significant change in the quantity of viable bacterial DNA, whilst culturability was significantly modulated, suggesting effects on bacterial growth phenotype beyond effects on xenophagic clearance. In either model, there was no evidence of host cell death to potentially explain the loss of culturability; for example, host cell ACTB protein and DNA levels were stable in response to any of the treatments used. Consistent with this, we showed in a recent study that at low MOIs similar to those used here, WCH-SK2 and another *S. aureus* strain caused no appreciable loss of human genomic signal as quantified by digital droplet PCR (Sun et al., 2023).

In the period immediately following infection, bafilomycin treatment caused a 2.1-fold increase in CFU recovered from cell lysates, presumably by reducing xenophagic stress on bacteria. This also occurred in a chronic infection model, exhibiting mostly non-culturable intracellular bacteria, where bafilomycin treatment appeared to facilitate a minor bacterial subpopulation returning to a culturable state. However, our results when modeling recurrence of a longer-term chronic infection showed that the potentiation (but not blockage) of autophagic flux was associated with a greater rate of bacterial escape and growth into the culture media, an effect that became evident 4–7 days after treatment. These findings suggest that in the chronic, non-culturable state, potentiation of autophagic/xenophagic flux imparts selective stress for a persister, high-growth phenotype. Our findings are also consistent with the report that stimulation of autophagy promoted *S. aureus* replication and host cell killing (Schnaith et al., 2007). Conversely, blockage of flux may select for low-growth phenotypes. Notably, there were no gross differences in colony morphology between conditions in the outgrowth assays, suggesting that the revertant clones were not SCVs but rather persister-type cells. The possible explanation for the interaction of autophagy with *S. aureus* in the context of flux blockers or potentiators is summarised in **Figure 4C**. Of note, xenophagy acting as a stress that induces a change in the growth phenotype is conceptually similar to reports of *S. aureus* adapting to autophagy/xenophagy in other host cell contexts (Schnaith et al., 2007; Wang et al., 2021). This has interesting implications for the mechanisms of recurrence in dormant infections, whereby dysfunction of autophagy in the host cell contributes to the regeneration of a culturable subpopulation.

Limitations of this study include the fact that xenophagic activity was measured by proxy via the flux of LC3A/B and p62/SQSTM1; this targeted analysis would miss potential changes in other pathways and proteins involved. Furthermore, in future studies, temporal and/or unbiased transcriptional regulation of autophagy markers also should be taken into account. Bacterial outgrowth was recorded as percentage of culture wells that became positive for growth; it was not possible to determine how many revertant events took place in any single well, and this could have varied between treatments. An interesting future direction related to this would be to examine the interactions of antibiotic treatments with autophagy modulation with respect to effects on intracellular bacterial growth phenotype and extracellular escape. In addition, the scope and clinical applicability of the observations is limited by the fact that a single *S. aureus* strain was used within a cell line model. This was done for consistency between readouts, and it is possible that other clinical *S. aureus* isolates might respond differently. The use of SaOS2-OY was also used for consistency, as donor variability is a potential confounder with primary cell models; however, this is the only validated human osteocyte-like cell line model described to date.

In conclusion, rifampicin alone and vancomycin treatment of human osteocyte-like cells caused some degree of autophagic flux inhibition, as did infection of these cells with *S. aureus*. Rifampicin and vancomycin affected the proportion of culturable bacteria but either did not affect the total population size or increased it. Targeted autophagic flux modulation in both the acute and chronic-like infection contexts tested was also capable of significantly affecting the proportion of culturable intracellular *S. aureus* but not the size of the intracellular bacterial pool. This suggests that a persister population of *S. aureus* escapes both antibiotic and autophagic/xenophagic stress by adopting a non-culturable growth phenotype. In contrast, in a long-term infection, potentiation of autophagy promotes *S. aureus* reactivation and escape from the host cell, whereas inhibition of autophagy appears to promote an intracellularly resident growth phenotype, suggesting that certain disruptions to host cell metabolism could be stimuli for recurrence of intracellular *S. aureus* infections.

## Data availability statement

The raw data supporting the conclusions of this article will be made available by the authors, without undue reservation.

## Ethics statement

Ethical approval was not required for the studies on humans in accordance with the local legislation and institutional requirements because only commercially available established cell lines were used.

## Author contributions

NG: Data curation, Formal analysis, Investigation, Methodology, Visualization, Writing – original draft, Writing – review & editing. SK: Methodology, Supervision, Validation, Writing – review & editing. LS: Funding acquisition, Writing – review & editing. DY: Funding acquisition, Methodology, Validation, Writing – review & editing. ER: Conceptualization, Data curation, Formal analysis, Investigation, Methodology, Resources, Supervision, Visualization, Writing – review & editing. GA: Conceptualization, Data curation, Funding acquisition, Methodology, Project administration, Resources, Supervision, Visualization, Writing – review & editing.

## Supplementary material

Supplementary Figure 1 Effects of antibiotics on SaOS2-OY viability. SAOS2-OY cells were generated as described in *Materials and Methods*. Percent cytotoxicity was determined using an LDH enzyme activity assay following 24 h of exposure to a dose range of **(A)** rifampicin and **(B)** vancomycin. Hollow data points represent doses within the range that osteocytes would be expected to be exposed to during clinical treatment *in vivo* and the dose range used for the remainder of this study. The 95% confidence interval is presented with the line of best fit, which for rifampicin was a linear regression and for vancomycin a one-phase association curve.
