# Supplementary figures and images for: Staphylococcus aureus persistence in osteocytes: weathering the storm of antibiotics and autophagy/xenophagy
Source: Front Cell Infect Microbiol. 2024 Jun 10;14:1403289. doi: 10.3389/fcimb.2024.1403289 (PMC11194354; doi:10.3389/fcimb.2024.1403289)

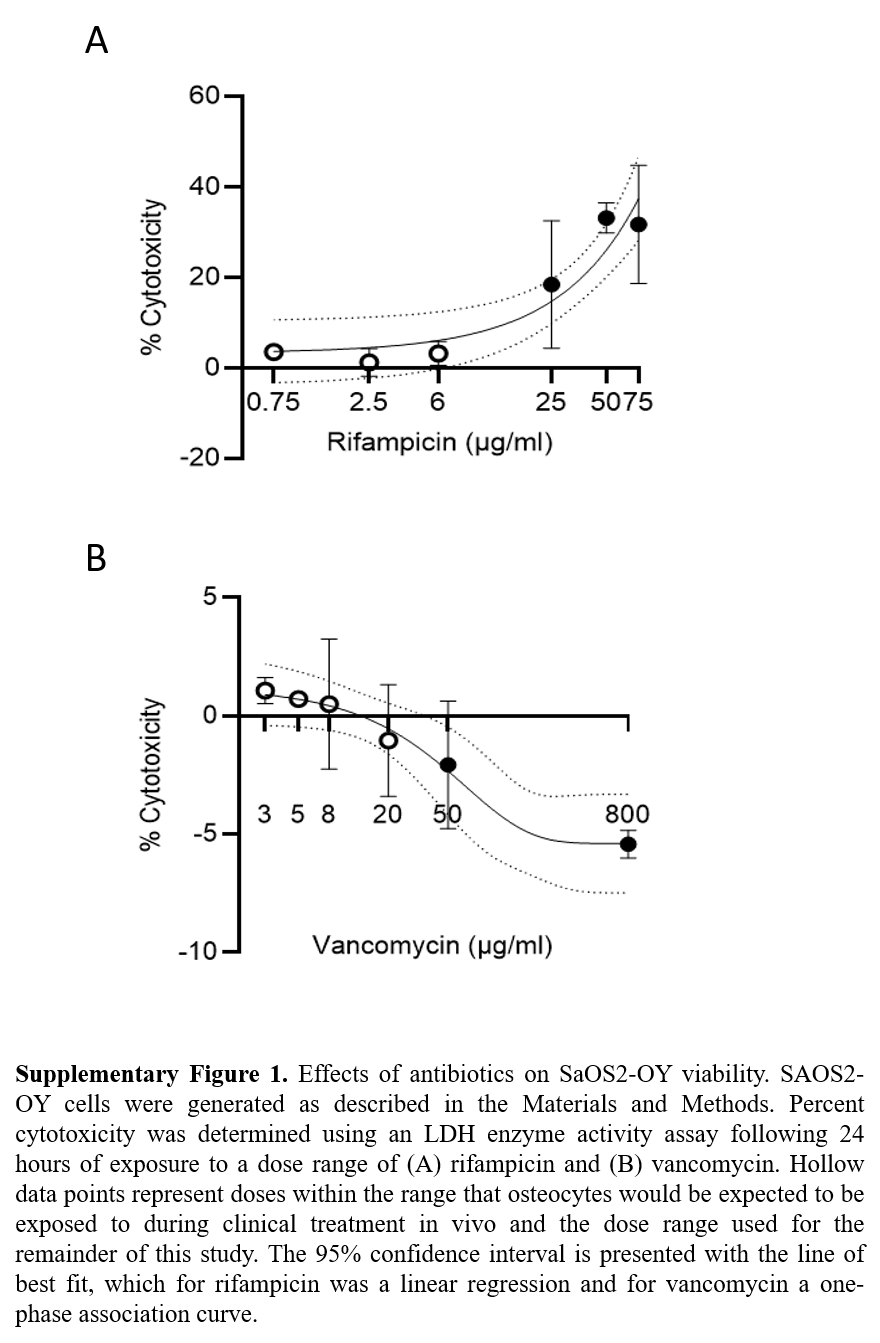

Supplement: Supplementary Figure 1 — Effects of antibiotics on SaOS2-OY viability. SAOS2-OY cells were generated as described in Materials and Methods. Percent cytotoxicity was determined using an LDH enzyme activity assay following 24 h of exposure to a dose range of (A) rifampicin and (B) vancomycin. Hollow data points represent doses within the range that osteocytes would be expected to be exposed to during clinical treatment in vivo and the dose range used for the remainder of this study. The 95% confidence interval is presented with the line of best fit, which for rifampicin was a linear regression and for vancomycin a one-phase association curve. [file Image_1.tif]

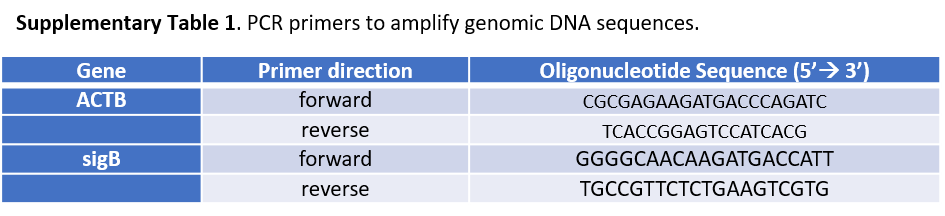

Supplement: Supplementary file 2 [file Image_2.tif]
